# Black Elderberry Exposure in Apparent Idiopathic Mild Acute Pancreatitis: A Case Report

**DOI:** 10.7759/cureus.112391

**Published:** 2026-07-10

**Authors:** Veronika Espinal, Krishna Rajesh, Eduardo Lazaro

**Affiliations:** 1 Internal Medicine, HCA Healthcare, Sarasota, USA; 2 Internal Medicine, HCA Florida Sarasota Doctors Hospital, Sarasota, USA

**Keywords:** acute pancreatitis, black elderberry, drug-induced pancreatitis, herbal supplement, medication reconciliation, sambucus nigra

## Abstract

Drug-induced acute pancreatitis is uncommon and remains difficult to confirm, particularly when the suspected exposure is an herbal or over-the-counter supplement. Black elderberry, derived from Sambucus nigra, is widely marketed for immune support, but published literature linking it to acute pancreatitis is limited. We report a 75-year-old woman with hypertension, hyperlipidemia, and prior cholecystectomy who presented with abrupt severe epigastric pain radiating to the back, diaphoresis, and lipase 3016 U/L. Computed tomography of the abdomen and pelvis on day 0 showed a normal-appearing pancreas without peripancreatic stranding, fluid collection, pancreatic ductal dilation, or biliary dilation. Triglycerides were 105 mg/dL, adjusted calcium was normal, bilirubin remained normal, and she denied alcohol use. Medication reconciliation revealed daily use of black elderberry extract along with chronic losartan, atenolol, niacin, aspirin, and latanoprost. Her course was consistent with mild acute pancreatitis. She improved with supportive care, early advancement of diet, and discontinuation of elderberry. Because occult biliary disease and medication co-exposure could not be definitively excluded, causality was conservatively classified as possible black elderberry-associated acute pancreatitis. This case reviews the importance of explicit supplement reconciliation in apparent idiopathic acute pancreatitis and the need for endoscopic ultrasound or magnetic resonance cholangiopancreatography after recovery in older adults when initial evaluation is unrevealing.

## Introduction

Acute pancreatitis is diagnosed when at least two of the following three features are present: characteristic abdominal pain, serum amylase or lipase at least three times the upper limit of normal, or imaging findings consistent with pancreatitis [1,2]. Computed tomography (CT) may be normal early in the disease course, particularly in mild acute pancreatitis, and imaging is not required when the clinical presentation and enzyme elevation establish the diagnosis [1,2].

Drug-induced acute pancreatitis (DIAP) is uncommon, with drugs estimated to account for approximately 0.1%-2% of acute pancreatitis cases [3]. Causality is often difficult to prove because rechallenge is usually unsafe, latency is often unclear, and alternative causes must be carefully excluded [3,4]. Herbal and over-the-counter (OTC) supplements create an additional diagnostic challenge because patients may not consider them medications and may not report them unless specifically asked.

Black elderberry, derived from Sambucus nigra, is commonly used for immune support. Published evidence linking black elderberry to acute pancreatitis is limited to rare case-level literature, including a prior report of pancreatitis associated with Sambucol, a commercial black elderberry preparation [5]. We present a case of black elderberry exposure in a 75-year-old woman with mild acute pancreatitis, normal early CT imaging, and no alcohol-related, hypertriglyceridemic, hypercalcemic, or obstructive biliary cause identified during the index admission.

## Case presentation

A 75-year-old woman with hypertension, hyperlipidemia, and a remote cholecystectomy in her 30s presented on day 0 with abrupt severe epigastric pain that began while walking. The pain was rated 9/10 to 10/10, radiated directly to the back, and was associated with profuse diaphoresis. She denied alcohol use, tobacco use, and recreational drug use.

She brought her home medications. When asked about supplements, she reported taking black elderberry daily before presentation, including the days preceding symptom onset. The exact dose and product formulation were unknown; the patient did not have the bottle.

On arrival, her temperature was 36.7 °C, heart rate was 67 beats/min, blood pressure was 144/67 mmHg, respiratory rate was 18 breaths/min, and oxygen saturation was 99% on room air. Physical examination showed focal epigastric tenderness without guarding or rebound. Cardiopulmonary and neurologic examinations were otherwise unremarkable.

Initial laboratory testing showed lipase 3016 U/L, white blood cell count 9.1 x 10^3/uL, sodium 134 mmol/L, creatinine 1.22 mg/dL, estimated glomerular filtration rate 46.3 mL/min/1.73 m2, glucose 131 mg/dL, triglycerides 105 mg/dL, calcium 8.9 mg/dL, albumin 3.6 g/dL, adjusted calcium 9.2 mg/dL, aspartate aminotransferase 43 U/L, alanine aminotransferase 28 U/L, alkaline phosphatase 84 U/L, and total bilirubin 1.0 mg/dL. On day 1, aspartate aminotransferase increased to 167 U/L and alanine aminotransferase increased to 172 U/L, while alkaline phosphatase and bilirubin remained within normal limits. On day 2, aspartate aminotransferase improved to 60 U/L, and alanine aminotransferase improved to 104 U/L. Key laboratory findings are summarized in Table 1.

**Table 1:** Key laboratory findings during hospitalization

Parameter	Day 0	Day 1	Day 2	Reference range	Interpretation
White blood cell count	9.1 x 10^3/uL	7.9 x 10^3/uL	6.6 x 10^3/uL	4.0-11.0 x 10^3/uL	No leukocytosis
Hemoglobin	13.4 g/dL	11.7 g/dL	10.9 g/dL	12.0-16.0 g/dL	Mild anemia by day 2
Lipase	3016 U/L	Not repeated	Not repeated	13-60 U/L	Greater than 3 times upper limit of normal
Triglycerides	105 mg/dL	Not repeated	Not repeated	<150 mg/dL	Hypertriglyceridemic pancreatitis unlikely
Sodium	134 mmol/L	135 mmol/L	137 mmol/L	136-145 mmol/L	Mild hyponatremia resolved
Creatinine	1.22 mg/dL	0.92 mg/dL	0.80 mg/dL	0.50-1.00 mg/dL	Mild renal dysfunction improved
Estimated glomerular filtration rate	46.3 mL/min/1.73 m2	64.9 mL/min/1.73 m2	76.8 mL/min/1.73 m2	>=60 mL/min/1.73 m2	Improved renal function
Calcium	8.9 mg/dL	8.3 mg/dL	8.9 mg/dL	8.6-10.2 mg/dL	No hypercalcemia
Albumin	3.6 g/dL	3.1 g/dL	3.1 g/dL	3.5-5.2 g/dL	Mild hypoalbuminemia
Adjusted calcium	9.2 mg/dL	9.0 mg/dL	9.6 mg/dL	8.6-10.2 mg/dL	Hypercalcemia not supported
Aspartate aminotransferase	43 U/L	167 U/L	60 U/L	0-35 U/L	Transient hepatocellular elevation
Alanine aminotransferase	28 U/L	172 U/L	104 U/L	0-35 U/L	Transient hepatocellular elevation
Alkaline phosphatase	84 U/L	104 U/L	94 U/L	35-104 U/L	No sustained cholestatic pattern
Total bilirubin	1.0 mg/dL	0.7 mg/dL	0.6 mg/dL	0.0-1.2 mg/dL	Biliary obstruction less likely

The patient met diagnostic criteria for acute pancreatitis based on characteristic epigastric pain radiating to the back and lipase 3016 U/L, which was greater than 3 times the upper limit of normal. Her course was classified as mild acute pancreatitis by the Revised Atlanta Classification because she had no persistent organ failure, pancreatic necrosis, peripancreatic fluid collection, or local or systemic complications [1].

**Figure 1:**
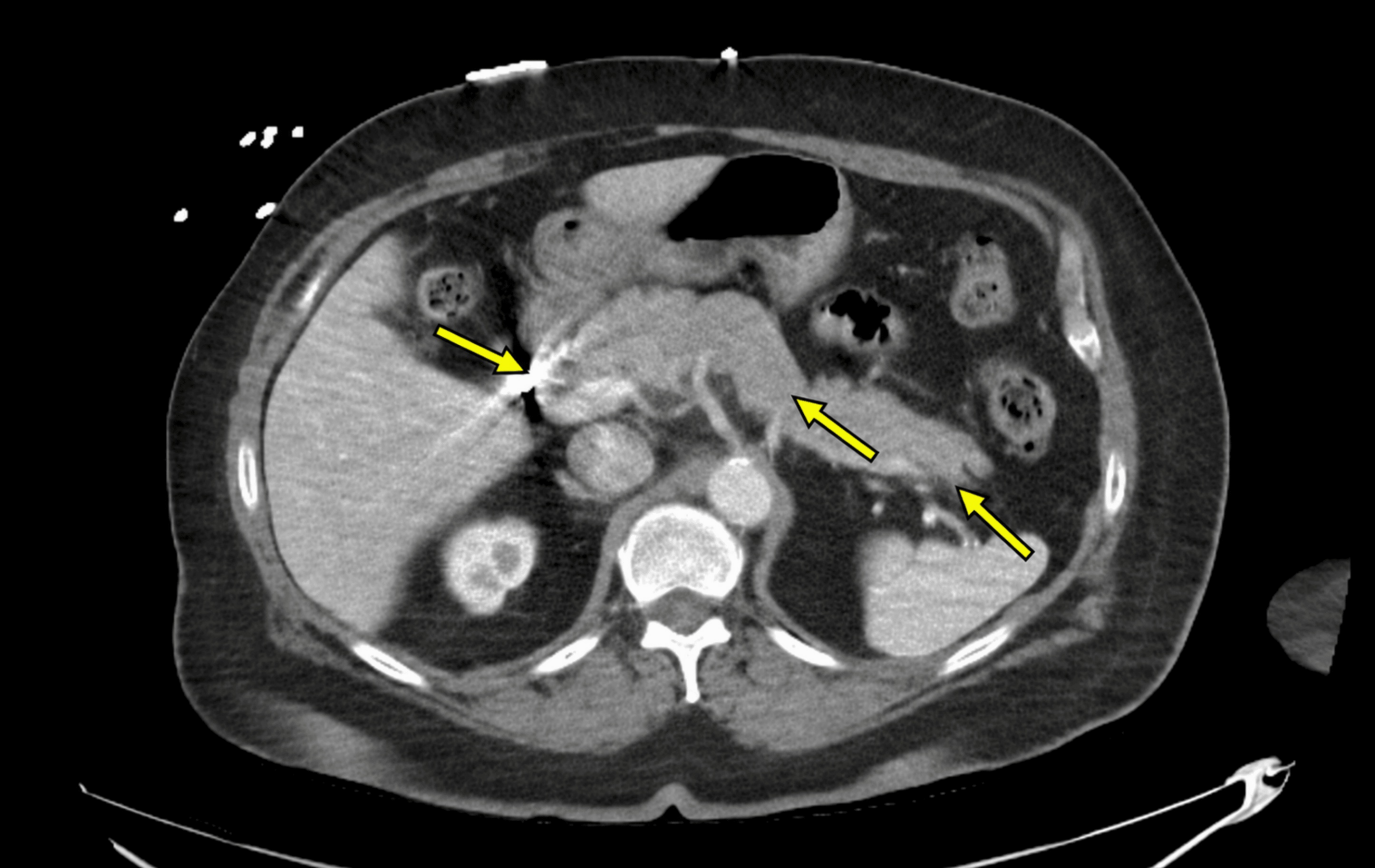
Axial contrast-enhanced CT abdomen, portal venous phase Yellow arrows highlight the pancreatic head/neck region, pancreatic body, and distal pancreatic body/tail region with adjacent peripancreatic fat planes. The arrows demonstrate preserved pancreatic enhancement and clean surrounding fat planes, without peripancreatic stranding, fluid collection, necrosis, or pancreatic ductal dilation. The figure illustrates the absence of CT-evident pancreatitis on day 0 despite clinical and biochemical diagnostic criteria for acute pancreatitis.

**Figure 2:**
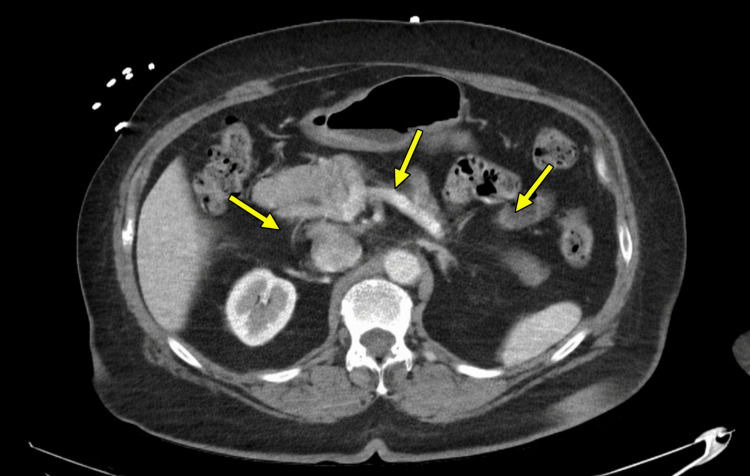
Axial contrast-enhanced CT abdomen, portal venous phase Yellow arrows highlight the pancreatic head/neck region, pancreatic body, and distal pancreatic body/tail region with adjacent peripancreatic fat planes. The pancreas demonstrates preserved enhancement without edema, peripancreatic inflammatory change, fluid collection, necrosis, or pancreatic ductal dilation. No common bile duct dilation is visualized in this image.

Contrast-enhanced CT of the abdomen and pelvis on day 0 showed a normal-appearing pancreas without peripancreatic stranding, pancreatic ductal dilation, peripancreatic fluid collection, necrosis, or biliary dilation. Incidental findings included hepatic steatosis and sigmoid diverticulosis. Representative CT images are shown in Figures 1-2.

Medication and supplement reconciliation was reviewed because the initial evaluation did not identify a clear common etiology. Her active home medications and supplements are summarized in Table 2.

**Table 2:** Medication and supplement reconciliation

Medication or supplement	Dose and frequency	Reconciliation status
Black elderberry extract	Daily use, exact dose and product formulation unknown	Reported active home supplement before symptom onset
Losartan potassium	50 mg PO daily	Active home medication
Atenolol	50 mg PO bedtime	Active home medication
Niacin extended-release	1000 mg PO bedtime	Active home medication
Aspirin delayed-release	81 mg PO daily	Active home medication
Latanoprost ophthalmic solution	1 drop each eye nightly	Active home medication

Alcohol-associated pancreatitis was unlikely based on the history. Hypertriglyceridemic pancreatitis was unlikely because triglycerides were 105 mg/dL. Hypercalcemia-associated pancreatitis was not supported because measured calcium and adjusted calcium remained within normal limits.

Biliary pancreatitis remained an important alternative consideration despite prior cholecystectomy. The patient had a transient alanine aminotransferase peak of 172 U/L, which can be seen after transient biliary obstruction or passage of a common bile duct stone. However, bilirubin remained normal, alkaline phosphatase was not elevated, and CT showed no biliary ductal dilation. Occult microlithiasis, biliary sludge, or a passed common bile duct stone could not be definitively excluded during the index admission without endoscopic ultrasound (EUS) or magnetic resonance cholangiopancreatography (MRCP).

Using the Naranjo adverse drug reaction probability scale [6], black elderberry-associated pancreatitis was classified conservatively as a possible adverse drug reaction, as shown in Table 3.

**Table 3:** Naranjo adverse drug reaction probability scale applied to black elderberry exposure

Question	Case finding	Score
Previous conclusive reports on this reaction?	Prior published case report of pancreatitis associated with a black elderberry preparation	+1
Event appeared after suspected agent was administered?	Patient reported daily black elderberry use before presentation	+2
Reaction improved after discontinuation?	Symptoms improved after supportive care and discontinuation of elderberry	+1
Reaction reappeared with readministration?	Rechallenge was not performed because intentional re-exposure was not clinically appropriate	0
Alternative causes possible?	Occult biliary disease and losartan co-exposure could not be definitively excluded	-1
Reaction reappeared with placebo?	Not applicable	0
Drug detected in toxic concentration?	Not applicable	0
Reaction worse with dose increase or improved with dose decrease?	Dose-response information was unavailable because the exact dose and product formulation were unknown	0
Similar prior reaction?	No prior similar reaction reported or documented	0
Objective evidence confirmed the adverse event?	Lipase 3016 U/L with typical epigastric pain radiating to the back	+1
Total	Possible adverse drug reaction	4

The score was limited by lack of rechallenge, unknown dose and product formulation, possible losartan co-exposure, and the need for follow-up EUS or MRCP to further exclude occult biliary or structural disease [6].

The patient was managed with moderate goal-directed intravenous lactated Ringer’s solution, analgesia, antiemetics, and clinical monitoring. Antibiotics were not administered because there was no evidence of infected necrosis, cholangitis, or another bacterial infectious source. She was restarted on a low-fat solid diet on day 1 and advanced as tolerated. Elderberry supplementation was discontinued. Her abdominal pain improved, she tolerated oral intake, and she was discharged with instructions to avoid elderberry.

Given this was the first episode of apparent idiopathic acute pancreatitis in an older adult, outpatient endoscopic ultrasound (EUS) was arranged after recovery, with magnetic resonance cholangiopancreatography (MRCP) as an alternative if EUS was unavailable or nondiagnostic. However, post-hospitalization EUS/MRCP results were not available to the authors at the time of manuscript revision. Therefore, occult microlithiasis, biliary sludge, a passed common bile duct stone, pancreatic ductal abnormality, or occult pancreatic neoplasm could not be definitively excluded.

## Discussion

This case highlights the importance of supplement reconciliation in apparent idiopathic acute pancreatitis. The patient met diagnostic criteria for acute pancreatitis based on classic epigastric pain radiating to the back and lipase 3016 U/L, despite a normal-appearing pancreas on CT performed on day 0. Early CT can be unrevealing in mild acute pancreatitis, and imaging is not required when characteristic pain and enzyme elevation establish the diagnosis [1,2]. Her clinical course was classified as mild acute pancreatitis because she had no persistent organ failure, pancreatic necrosis, peripancreatic fluid collection, or local or systemic complications [1].

Drug-induced acute pancreatitis (DIAP) is uncommon, accounting for approximately 0.1%-2% of acute pancreatitis cases [3]. Establishing causality is difficult because rechallenge is usually unsafe, latency may be unclear, and alternative etiologies must be excluded [3,4]. Herbal and over-the-counter supplements make this assessment even more challenging because patients may not consider them medications. In this case, black elderberry exposure was identified only because the patient brought her home medications and supplements for review.

Black elderberry-associated pancreatitis appears to be rare, and no reliable population-level incidence estimate exists. The published signal remains limited to case-level literature, including a prior report of pancreatitis associated with Sambucol, a commercial black elderberry preparation [5]. Our case adds a clinically distinct presentation involving an older post-cholecystectomy patient with mild acute pancreatitis, normal early CT imaging, and a suspected supplement exposure that could have been missed without deliberate supplement reconciliation.

Losartan was included as a possible confounder rather than a favored primary cause. Losartan-associated pancreatitis has been reported, but the evidence remains limited and rare, with no established incidence estimate [7]. In this patient, losartan was a chronic therapy without documented recent initiation or dose change. However, medication co-exposure still matters in DIAP assessment. Although a specific black elderberry-losartan interaction has not been established, an additive or synergistic susceptibility effect cannot be excluded. For that reason, black elderberry should be interpreted as a possible contributor rather than a confirmed independent cause.

Biliary pancreatitis also remained an important alternative consideration despite prior cholecystectomy. The transient alanine aminotransferase elevation to 172 U/L raised the possibility of transient biliary obstruction or a passed common bile duct stone. However, bilirubin remained normal, alkaline phosphatase was not elevated, and CT showed no biliary ductal dilation. These findings made persistent obstruction less likely but did not fully exclude microlithiasis, biliary sludge, or a passed stone.

A first episode of apparent idiopathic acute pancreatitis in an older adult should prompt structural evaluation after recovery. Endoscopic ultrasound (EUS) and magnetic resonance cholangiopancreatography (MRCP) are complementary tests in idiopathic acute pancreatitis. A systematic review and meta-analysis found a higher diagnostic yield with EUS than MRCP overall, particularly for biliary disease and chronic pancreatitis [8]. Older age is also clinically relevant because pancreatic cancer can present with acute pancreatitis. In a Danish nationwide cohort study, age greater than 50 years was a predictor of underlying pancreatic cancer after acute pancreatitis [9]. In this case, outpatient EUS was arranged after recovery, with MRCP as an alternative if EUS was unavailable or nondiagnostic. However, post-hospitalization EUS/MRCP results were not available to the authors at the time of manuscript revision. Therefore, occult biliary, structural, or neoplastic causes could not be definitively excluded.

Management aligned with contemporary acute pancreatitis principles. The patient improved with moderate goal-directed intravenous fluids, analgesia, antiemetics, early oral feeding, and discontinuation of black elderberry. Antibiotics were avoided because there was no evidence of infected necrosis, cholangitis, or another bacterial source. The WATERFALL randomized trial found that aggressive early fluid resuscitation increased fluid overload without improving clinical outcomes, supporting a more measured approach [10].

The main limitation is that causality cannot be proven. Rechallenge was not performed because intentional re-exposure after suspected pancreatitis would be unsafe. The exact dose and product formulation of black elderberry were unknown, which limited dose-response assessment. Post-hospitalization EUS/MRCP results were not available to the authors at the time of manuscript revision, so occult biliary disease, structural pancreatic disease, or pancreatic neoplasm could not be definitively excluded. Despite these limitations, this case reinforces a practical diagnostic lesson: herbal and over-the-counter supplements should be specifically reconciled in apparent idiopathic acute pancreatitis, especially when standard etiologic testing is unrevealing.

## Conclusions

Black elderberry-associated acute pancreatitis is rare and supported only by limited case-level literature. This case does not prove causation, but it documents black elderberry exposure in an older adult with mild acute pancreatitis and no alcohol-related, hypertriglyceridemic, hypercalcemic, or obstructive biliary cause identified during the index admission. Chronic losartan exposure and occult biliary disease remain possible confounders, and supplement-drug co-exposure may have contributed to susceptibility. A normal early CT scan should not override characteristic pain and marked lipase elevation. Clinicians should ask specifically about herbal and OTC supplements, document all active prescription and nonprescription exposures, discontinue suspected agents, and arrange EUS or MRCP after recovery in older adults with apparent idiopathic acute pancreatitis.
